# Cardiovascular risk status of Afro-origin populations across the spectrum of economic development: findings from the Modeling the Epidemiologic Transition Study

**DOI:** 10.1186/s12889-017-4318-4

**Published:** 2017-05-12

**Authors:** Lara R. Dugas, Terrence E. Forrester, Jacob Plange-Rhule, Pascal Bovet, Estelle V. Lambert, Ramon A. Durazo-Arvizu, Guichan Cao, Richard S. Cooper, Rasha Khatib, Laura Tonino, Walter Riesen, Wolfgang Korte, Stephanie Kliethermes, Amy Luke

**Affiliations:** 1 Public Health Sciences, Stritch School of Medicine, Maywood, IL USA; 2 Solutions for Developing Countries, University of the West Indies, Mona, Kingston, Jamaica; 3 Department of Medicine, Kwame Nkrumah University of Science and Technology, Kumasi, Ghana; 4 Ministry of Health, Republic of Seychelles, Seychelles, Seychelles; 5 Institute of Social & Preventive Medicine, Laussanne University Hospital, Lausanne, Switzerland; 6 Research Unit for Exercise Science and Sports Medicine, University of Cape Town, Cape Town, South Africa; 7 Center for Laboratory Medicine, Canton Hospital, St. Gallen, Switzerland; 8 Department of Orthopaedics and Rehabilitation, University of Wisconsin School of Medicine and Public Health, Madison, WI USA

**Keywords:** African diaspora, Human development index, Cardiovascular risk

## Abstract

**Background:**

Cardiovascular risk factors are increasing in most developing countries. To date, however, very little standardized data has been collected on the primary risk factors across the spectrum of economic development. Data are particularly sparse from Africa.

**Methods:**

In the Modeling the Epidemiologic Transition Study (METS) we examined population-based samples of men and women, ages 25–45 of African ancestry in metropolitan Chicago, Kingston, Jamaica, rural Ghana, Cape Town, South Africa, and the Seychelles. Key measures of cardiovascular disease risk are described.

**Results:**

The risk factor profile varied widely in both total summary estimates of cardiovascular risk and in the magnitude of component factors. Hypertension ranged from 7% in women from Ghana to 35% in US men. Total cholesterol was well under 200 mg/dl for all groups, with a mean of 155 mg/dl among men in Ghana, South Africa and Jamaica. Among women total cholesterol values varied relatively little by country, following between 160 and 178 mg/dl for all 5 groups. Levels of HDL-C were virtually identical in men and women from all study sites. Obesity ranged from 64% among women in the US to 2% among Ghanaian men, with a roughly corresponding trend in diabetes. Based on the Framingham risk score a clear trend toward higher total risk in association with socioeconomic development was observed among men, while among women there was considerable overlap, with the US participants having only a modestly higher risk score.

**Conclusions:**

These data provide a comprehensive estimate of cardiovascular risk across a range of countries at differing stages of social and economic development and demonstrate the heterogeneity in the character and degree of emerging cardiovascular risk. Severe hypercholesterolemia, as characteristic in the US and much of Western Europe at the onset of the coronary epidemic, is unlikely to be a feature of the cardiovascular risk profile in these countries in the foreseeable future, suggesting that stroke may remain the dominant cardiovascular event.

## Introduction

The magnitude and rate of change in cardiovascular disease (CVD) risk among populations has become increasingly well understood over the last two decades. The steep, monotonic downward course of both coronary heart disease and stroke observed in most industrialized countries since the 1970’s has resulted in a decline in mortality rates in countries like Finland and the US [1]. At the same time, an enormous surge in CVD mortality has been documented in Russia and other regions from the former USSR, and CVD risk status is increasing markedly in China [2, 3].

The transition to industrialized consumer societies has been invariably accompanied by rising CVD risk and a key challenge in global health is to prevent a new wave of the CVD epidemic from engulfing countries undergoing this transition [4]. The investment in large scale data projects such as the Global Burden of Disease (GBD) has created an initial – albeit fragmentary -description of trends in CVD risk in developing countries [5]. While this new emphasis on temporal trends in CVD has greatly strengthened the position of those in the public health community who argue in favor of population-wide prevention, many important questions remain unanswered. The vast majority of developing countries lack both adequate vital records systems to monitor mortality and representative survey data to assess CVD risk status. The current level of CVD burden in these countries, and the projections for future trends, must therefore be based on local surveys, inferences from other countries in the region with appropriate data or some other form of imputation. Of all the regions in the world, sub –Saharan Africa has by far the weakest public health infrastructure, and virtually all published estimates of CVD risk are based on extremely limited empirical data [6, 7].

Paradoxically, the CVD burden of persons of African descent living in US, and to a lesser extent in the Caribbean and the UK, has been the subject of intense scrutiny over the last half century. Black Americans have approximately 50% higher rates of hypertension, and as a consequence suffer significantly increased mortality from stroke [8, 9]. On a global scale, however, the apparent excess hypertension risk seen in black Americans is an artifact of the comparison to US whites; in fact, blood pressures in Caribbean blacks are similar to those among US whites and hypertension rates in West Africa generally low [10]. While it is now possible to place the hypertension risk of populations of African origin within the international context, to our knowledge no comparative studies of other aspects of CVD risk, particularly serum lipids, has yet been undertaken. In this report we describe both serum lipid levels and other key measures of CVD risk in five populations across the African diaspora that span the socio-economic spectrum.

## Methods

### Sampling design and participant recruitment

Twenty-five hundred adults, ages 25–45, were enrolled in the Modeling the Epidemiologic Transition Study (METS) between January 2010 and December 2011. A detailed description of the study protocol and sample size calculations has been previously published [11]. In brief, five hundred participants, approximately 50% of whom are female, were enrolled in each of five study sites: rural Ghana, peri-urban South Africa, mixed urban/rural Seychelles, urban Jamaica and metropolitan Chicago. All participants, with the exception of the Seychelles, were of African descent. Probability sampling was not employed to represent urban-rural populations within each site. However, the sample is diverse in terms of types of communities and includes rural, peri-urban, and urban sites. Further, the study sites were selected to represent a range of body sizes, i.e., the mean (Body Mass Index) BMI of adults from the study sites vary from a low of about 24 kg/m^2^ in rural Nkwantakese (Ghana) to a high of 31 kg/m^2^ in suburban Maywood (USA) [11]. The study sites also represent a broad range of social and economic development as defined by the UN Human Development Index (HDI) 2010: i.e., Ghana as a low HDI country, South Africa as middle, Jamaica and the Seychelles as high, and the US as a very high HDI country [12].

Exclusion criteria included individuals with infectious diseases, including HIV-positive individuals, and pregnant or lactating women, as well as persons with conditions preventing normal physical activities, e.g. lower extremity disability. In Ghana, a simple random sample was generated for the age-range of the study from the population census for the rural town of Nkwantakese. In both Seychelles and South Africa sex- and age-stratified random samples were generated from their respective national censuses. In Kingston, Jamaica, districts were randomly sampled; beginning from a fixed point in each district (e.g., the north-west corner), and door-to-door recruitment was then carried out. Similarly, in Maywood, IL, USA, all city blocks in the community were randomized and door-to-door recruitment was conducted.

The protocol for METS was approved by the Institutional Review Board of Loyola University Chicago, IL, USA; the Committee on Human Research Publication and Ethics of Kwame Nkrumah University of Science and Technology, Kumasi, Ghana; the Research Ethics Committee of the University of Cape Town, South Africa; The Ministry of Health and Social Development Public Health Department, Mahé, Republic of Seychelles; the Ethics Committee of the University of the West Indies, Kingston, Jamaica; and the Health Sciences Institutional Review Board of the University of Wisconsin, Madison, WI, USA. Written informed consent was obtained from all participants in the 5 participating countries.

### Measurements

All measurements were made at outpatient clinics located in the communities. We developed and used manuals and training workshops for all study personnel to ensure high quality data. Standardized data collection forms were used during the data collection process. Once collected, all data were entered into the same database and data range checks were employed as additional quality control measures.

Demographic characteristics including education, employment, and type of labor (manual or not) were self-reported using standardized questionnaires. Physical activity was measured using an accelerometer (Actical, Phillips Respironics, Bend, OR, USA). Smoking status was self-reported and was defined as smoking cigarettes, cigars, or pipes regularly (at least one per day) for at least one year.

Weight (kg) and height (cm) measurements were made on all participants while wearing light clothing and no shoes. Weight was measured to the nearest 0.1 kg using the same model standard calibrated balance at all five sites (Seca 770, Hamburg, Germany). Height was measured to the nearest 0.1 cm using a stadiometer (e.g. *Invicta* Stadiometer, Invicta, London, UK) with the participant’s head held in the Frankfort plane. Waist circumference was measured to the nearest 0.1 cm at the umbilicus and hip at the point of maximum extension of the buttocks. BMI was calculated as kg/m^2^.

Body composition was estimated by bioelectrical impedance analysis (BIA) with the use of a single-frequency (50 kHz) impedance analyzer (model BIA 101Q; RJL Systems, Clinton Township, MI). A tetrapolar placement of electrodes was used on the right hand and foot. Fat-free mass and fat mass were estimated from measured resistance by using an equation developed and validated in the METS cohorts [13].

Blood pressure was measured using the protocol of our ongoing international hypertension studies [14]. Systolic and diastolic blood pressure and pulse were measured using the Omron Automatic Digital Blood Pressure Monitor (model HEM-747Ic, Omron Healthcare, Bannockburn, IL, USA). With the antecubital fossa at heart level, three measurements were made at each of two time points separated by approximately 60 min for a total of six measurements.

### Biochemical measures

Participants were asked to fast from the evening prior to the baseline clinic examination (10 to 12 h). Study personnel were trained and given clear instructions to draw blood only if the participant had fasted. In the event of a non-fasting participant, blood was not drawn, and the participant was asked to fast and return on the following examination day. Fasting blood samples were drawn for analysis of blood sugar, total cholesterol (TC), triglycerides (TG), and HDL-cholesterol (HDCL-C). LDL-cholesterol (LDL-C) was calculated using the Friedewald equation (LDL-C = TC- (HDL-C + TG/5) [15]. The blood samples were processed and plasma or serum separated within two hours of collection and stored at -80 °C in the laboratory at each study site. All assays were conducted at the Zentrum fϋr Labormedizin, Leiter Klinische Chemie und Hämatologie, St. Gallen, Switzerland. Serum lipids were all measured using the endpoint method (DXC analyzer, Beckman Coulter). Type I Diabetes (referred to as diabetes) was defined as blood glucose levels ≥140 mg/dl in Ghana or ≥125 mg/dl in all other sites.

### Statistical analyses

Participant characteristics and CVD risk factors were summarized using means ± standard deviations (SD) and proportions. CVD risk scores were calculated based on the Framingham risk equations developed using the Joint National Committee (JNC-V) categorization of blood pressure and the National Cholesterol Education Program (NCEP) categorization of TC and LDL-C [16]. Briefly, β-coefficients from sex-specific Cox proportional hazards models for selected CVD risk factors, age, blood pressure, cigarette use, diabetes status, LDL-C (or TC), and HDL-C, were applied to the corresponding measurements obtained on METS participants. Statistical analyses was performed using Stata (version 12, College Station, TX).

## Results

A total of 2506 participants were recruited into METS. Blood samples were analyzed for 94% (*n* = 2364) of recruited participants. Participants with missing blood analysis were excluded from these analyses (5.7%). The majority of missing data came from Jamaica (missing among 103 of 500 participants). Excluded participants (who had missing data) were similar in age (*p*-value > 0.05) but more likely to be males (*p*-value < 0.001) compared to participants included in the analysis.

The descriptive characteristics of the participants, by site, are presented in Tables 1 and 2. Mean age was similar across sites and ranged from 33.07 ± 5.98 years among women in South Africa to 36.50 ± 5.12 years among men in the Seychelles. Given the focus of the parent METS study on obesity, large variations in BMI are observed by site and by sex. BMI was lowest among Ghanaian men (22.23 ± 2.68 kg/m^2^) and South African men (22.44 ± 4.27 kg/m^2^) and was highest among women in the US (34.07 ± 8.83 kg/m^2^). Similarly, mean percent body fat was lowest among Ghanaian men (16.18 ± 5.83%) and highest among US women (44.57 ± 6.53%). Among both men and women, Ghanaians were 6 cm shorter than the African Americans, likely reflecting deficient nutrient intake before puberty. South African women and men were likewise 4–6 cm shorter than their US counterparts. Ghanaian women were the least educated (7.53 ± 4.12 years) while women in the US (13.82 ± 2.61 years) were the most educated.

**Table 1:** Male characteristics by site – mean ± SD

	Ghana	South Africa	Jamaica	Seychelles	United States
Sample size	206	231	153	229	236
Age (y)	34.60 ± 6.73	33.71 ± 5.59	34.03 ± 5.94	36.50 ± 5.12	35.60 ± 6.23
Weight (kg)	63.57 ± 9.13	65.58 ± 13.58	73.15 ± 14.96	80.13 ± 15.98	92.77 ± 24.82
Height (cm)	168.99 ± 6.63	170.87 ± 6.34	176.02 ± 6.68	173.87 ± 6.16	176.57 ± 6.59
Body Mass Index (kg/m^2^)	22.23 ± 2.68	22.44 ± 4.27	23.58 ± 4.47	26.47 ± 4.92	29.70 ± 7.53
Waist Circumference (cm)	77.04 ± 10.52	80.91 ± 11.47	80.28 ± 12.08	89.40 ± 11.77	97.16 ± 21.50
Hip Circumference (cm)	91.80 ± 10.85	94.59 ± 8.43	95.12 ± 9.28	102.74 ± 9.56	109.21 ± 15.89
Fat-free Mass (kg)	53.02 ± 5.11	50.63 ± 6.26	57.48 ± 6.93	59.64 ± 7.81	63.29 ± 9.71
Fat Mass (kg)	10.72 ± 5.25	14.96 ± 8.33	15.67 ± 9.19	20.75 ± 9.82	29.48 ± 16.56
% Body Fat	16.18 ± 5.83	21.66 ± 6.91	20.13 ± 7.43	24.67 ± 7.45	29.73 ± 9.01
Plasma Glucose (mg/dL)	100.92 ± 11.67	84.99 ± 13.82	95.35 ± 9.12	107.02 ± 37.20	105.79 ± 34.14
Systolic Blood Pressure (mmHg)	118.92 ± 13.06	128.96 ± 17.07	121.53 ± 12.84	122.63 ± 14.52	127.91 ± 14.55
Diastolic Blood pressure (mmHg)	68.50 ± 11.41	79.59 ± 13.11	71.22 ± 11.13	74.95 ± 11.39	81.04 ± 12.11
MVPA* (1-min bouts)	46.9 ± 24.6	55.5 ± 34.6	29.6 ± 23.3	35.9 ± 24.3	33.1 ± 34.7
Education (y)	9.21 ± 3.95	9.52 ± 2.57	10.64 ± 2.17	13.04 ± 2.33	12.73 ± 1.61
Employed, n (%)	180 (98.36%)	211 (92.10%)	199 (92.13%)	209 (98.12%)	190 (84.07%)
Manual Laborer, n (%)	108 (60.00%)	203 (90.63%)	120 (60.91%)	97 (54.49%)	136 (66.67%)

*MPVA moderate vigorous physical activity by accelerometer

**Table 2:** Female characteristics by site– mean ± SD

	Ghana	South Africa	Jamaica	Seychelles	United States
Sample size	288	267	244	265	245
Age (y)	34.03 ± 6.63	33.07 ± 5.98	34.72 ± 6.18	35.81 ± 6.04	35.00 ± 6.28
Weight (kg)	63.59 ± 13.12	81.97 ± 22.27	78.54 ± 18.58	72.09 ± 17.25	91.71 ± 24.40
Height (cm)	157.96 ± 5.71	160.15 ± 6.30	163.24 ± 6.58	161.37 ± 6.52	164.00 ± 6.20
Body Mass Index (kg/m^2^)	25.50 ± 5.20	31.90 ± 8.18	29.49 ± 6.75	27.64 ± 6.23	34.07 ± 8.83
Waist Circumference (cm)	84.20 ± 12.48	96.90 ± 16.44	91.95 ± 13.80	87.91 ± 12.37	101.88 ± 19.56
Hip Circumference (cm)	100.23 ± 13.26	114.15 ± 15.66	107.90 ± 11.62	104.42 ± 12.40	117.05 ± 16.06
Fat-free Mass (kg)	40.80 ± 5.33	45.19 ± 8.01	46.45 ± 7.11	43.92 ± 7.09	49.57 ± 8.58
Fat Mass (kg)	22.79 ± 8.50	36.77 ± 14.85	32.14 ± 12.09	28.17 ± 11.16	42.27 ± 16.36
%Body Fat	34.82 ± 6.07	43.36 ± 6.63	39.71 ± 6.09	37.81 ± 6.72	44.57 ± 6.53
Plasma Glucose (mg/dL)	99.80 ± 12.47	83.06 ± 28.78	90.91 ± 9.24	95.28 ± 17.95	100.56 ± 35.36
Systolic Blood Pressure (mmHg)	110.47 ± 15.17	118.20 ± 18.62	115.25 ± 14.74	112.26 ± 26.54	117.44 ± 16.16
Diastolic Blood pressure (mmHg)	66.18 ± 11.44	76.30 ± 11.78	72.10 ± 11.38	72.70 ± 26.36	79.60 ± 13.21
^a^MVPA (1-min bouts)	25.9 ± 16.7	22.0 ± 16.3	19.5 ± 15.3	22.9 ± 14.7	15.1 ± 17.7
Education (y)	7.53 ± 4.12	10.05 ± 2.13	10.78 ± 2.02	12.97 ± 2.38	13.82 ± 2.61
Employed (%)	241 (89.59%)	217 (81.89%)	165 (72.05%)	222 (95.28%)	199 (83.61%)
Manual Laborer (%)	231 (89.88%)	207 (89.61%)	130 (68.42%)	47 (25.13%)	65 (29.82%)

^a^ *MPVA* moderate vigorous physical activity by accelerometer

**Fig. 1:**
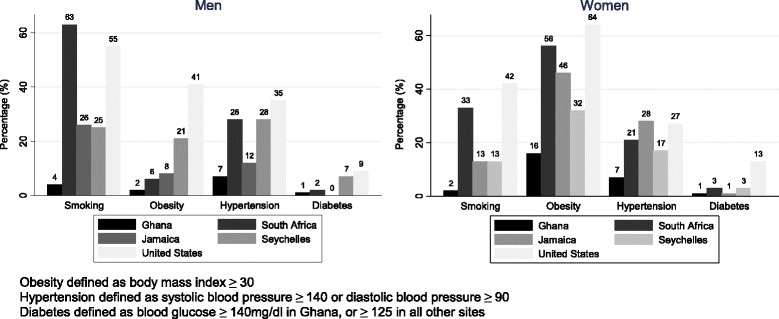
Prevalence of smoking, obesity, hypertension, and diabetes by Site and Sex

Prevalence of CVD risk factors varied markedly by study site (Fig. 1). Rates of smoking, obesity, hypertension, and diabetes were all markedly higher among US men and women (highest HDI), compared to Ghanaian men and women (lowest HDI). However, rates among countries with medium HDI did not show a clear pattern. Among men, obesity prevalence ranged from 1.5% in Ghana to 41% in US. In Ghana, Seychelles, and the US obesity prevalence was shifted upward by an additional 10–20% in women compared to men. However in South Africa and Jamaica a much more prominent sexual dimorphism was observed, with 10-fold higher rates in South African women than their male counterparts and 5-fold higher in women than men in Jamaica. The geographic variation in diabetes was not consistent with trends in obesity, with much lower rates observed in South Africa and Jamaica than might be anticipated, especially among women. Hypertension prevalence did, however, closely mirror the obesity rates across sites, with exception of men in South Africa where high blood pressure was disproportionately higher.

Total cholesterol levels were substantially lower among men outside the US (Table 3), and this was reflected mainly in lower LDL-C. The distribution of mean HDL-C levels was more complex, however. Among men HDL-C levels in Ghana, Jamaica and the Seychelles clustered in the mid-40’s mg/dl; HDL-C levels were highest in South Africa (57 mg/dl), followed by the US (50 mg/dl). Among women all the non-US sites had mean values of 46–48 mg/dl, while in the US HDL-C approached 52 mg/dl. Triglyceride levels were highest in the US and Seychelles and South Africa among men, and the US among women. There was no apparent inverse relationship between HDL-C and triglycerides by site, in fact among women both HDL-C and triglycerides were highest in the US.

**Table 3:** Serum Lipids (mg/dl) by site and sex, mean ± SD

	N	Ghana	N	South Africa	N	Jamaica	N	Seychelles	N	United States
Men										
Cholesterol (mg/dL)	206	154.54 ± 35.29	231	154.94 ± 40.46	153	155.32 ± 32.61	231	175.75 ± 35.69	236	181.75 ± 39.20
Triglyceride (mg/dL)	206	88.15 ± 45.90	230	96.08 ± 62.70	153	70.74 ± 32.16	231	97.58 ± 77.38	236	95.40 ± 57.15
HDL-C (mg/dL)	205	44.67 ± 16.36	230	56.67 ± 19.76	153	47.11 ± 12.62	128	47.39 ± 13.68	235	50.06 ± 14.91
LDL-C (mg/dL)	205	92.16 ± 30.14	228	82.62 ± 31.89	153	94.13 ± 29.70	128	112.66 ± 34.79	235	112.36 ± 34.43
Women										
Cholesterol (mg/dL)	288	166.11 ± 34.07	268	159.60 ± 33.22	244	166.96 ± 33.81	265	165.66 ± 34.51	245	178.25 ± 38.67
Triglyceride (mg/dL)	288	77.25 ± 34.86	268	76.18 ± 37.11	244	74.28 ± 38.39	265	64.48 ± 35.70	245	96.95 ± 61.75
HDL-C (mg/dL)	288	47.20 ± 13.17	268	46.22 ± 16.54	244	46.14 ± 11.80	178	47.85 ± 12.16	244	51.71 ± 14.87
LDL-C (mg/dL)	288	103.38 ± 29.38	268	98.12 ± 27.79	244	105.85 ± 28.54	178	109.08 ± 32.88	244	107.28 ± 33.11

*HDL-C* HDL-cholesterol, *LDL-C* LDL-cholesterol

**Fig. 2:**
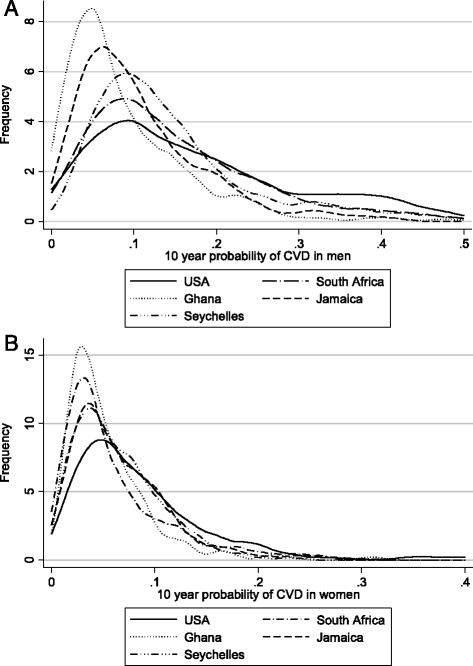
CVD risk scores based on the Framingham risk equations among the men (**a**, *N* = 1055) and women (**b**, *N* = 1309)

A continuous risk score based on the Framingham model was used to generate summary estimates of CVD risk (Fig. 2a, b). Substantial overlap existed for women in the cross-site comparison, although US women clearly had a higher risk score distribution. Among men, on the other hand, more separation of the risk score distributions was observed, with the rank order, low to high, − Ghana, Jamaica, Seychelles, South Africa, and finally the US.

## Discussion

The data presented in this report depict a heterogeneous pattern of CVD risk in populations of African origin. Overall, the prevalence of CVD risk factors are much lower in Ghana (lowest HDI) compared to the United States (highest HDI), while in sites with a medium HDI prevalence’s fall between those observed in Ghana and the United States. The summary CVD risk, as provided by the Framingham model, shows a clear trend toward higher levels in men across the spectrum of economic development, however in women there is broad over-lap in the non-US groups. The largest contrasts in prevalence rates are seen in obesity and hypertension, ranging from 2 to 64% for the former and 7 to 35% for the latter. Diabetes prevalence ranged from less than 1 % in Ghanaian and Jamaican men to 13% in US women, roughly in parallel with obesity rates. Data from the GBD study noted high rates of obesity among children in North Africa and the Caribbean suggesting a rise in future obesity rates among these populations [17].

Among two groups the prevalence rates for the CVD risk factors are not consistent with expectation; for reasons that are unclear Jamaican women had much lower diabetes rates than would be anticipated on the basis of the BMI in this study group, and in South Africa men had hypertension rates similar to the US, despite being lean and active. As is widely recognized, diabetes is now common in the Caribbean and it is possible that our recruitment strategy led to a biased sample [18]. We have previously suggested that the hypertension rates in South African men may reflect psychosocial pressures and racial discrimination [19]. Our sample is focused on populations of African origin from different regions of the world (the US, the Caribbean, and Africa). Part of the observed heterogeneity could be explained by the differences in economic development. CVD risk factors are expected to be higher in more developed countries that have undergone an epidemiologic transition which has been demonstrated using GBD data [20]. However, the observed heterogeneity in risk factors could also be explained by regional, cultural, and healthcare system differences, in addition to differences due to difference levels of economic development. For example, smoking rates remain lower in Ghana compared to the US despite it being more developed.

LDL-C levels were substantially lower among men outside the US. In the US, mean LDL-C levels were similar to those reported by the National Health and Nutrition Examination Surveys among Non-Hispanic Black men [21]. Among the serum lipid fractions the pattern of HDL-C is the most notable finding. By default, given the rich survey literature, as a standard reference it is normally expected that men will have HDL-C levels 10 mg/dl lower than women [22, 23]. Higher HDL-C in African American men has been well documented, and a small male: female gradient is observed [24]. It is surprising, however, that HDL levels were higher in men than women in Jamaica and South Africa, while being approximately the same among men compared to women in Ghana, the Seychelles, and the US (Table 3). These data challenge the construct that European populations represent the biological norm. It must be noted, however, that the marked gender dimorphism in obesity prevalence in Jamaica and South Africa is likely accounting the relatively low HDL levels in women from those sites. Generalizing from this five study samples, it appears that in populations of African descent sex-specific HDL levels are similar.

The data presented here confirm the heterogeneity of CVD risk in Afro-origin populations. As expected, hypertension is common outside of rural communities. Obesity is also widespread particularly among women, and a severe wave of diabetes can be anticipated in the very near future for all these communities. This has been reported in the literature for the African region and projections suggest a 110% absolute increase in the prevalence of diabetes between 2013 and 2035 [25]. The evolution of CVD in the period of the peak epidemic in the US and Europe was driven by high serum cholesterol levels and widespread use of tobacco [26]. Our data suggest that both of those aspects of CVD risk will not occur in Africa and the Caribbean, certainly within the coming decades.

A narrow difference in HDL-C between black men and women in the US compared to whites has long been noted. These data confirm near equality of HDL-C in both genders across these populations, with modestly higher levels in men from Jamaica and especially South Africa. The gender dimorphism in obesity likely provides an explanation for the finding in South Africa. The consistency in HDL-C levels across the remaining groups demonstrates that, contrary to what is observed in European populations, very little gender-specific effects on HDL-C are found in groups from sub-Saharan Africa.

The Prospective Urban Rural Epidemiology (PURE) study is one of the few cohorts comparing CVD risk by country income group using standardized measures and complement the results presented in this paper indicating lower CVD risk in low- and middle- income countries compared to high- income countries [27].

Our study is one of the few studies examining CVD risk across multiple sites in Africa, Jamaica, and the United States. High quality data from African countries that are comparable with other regions of the world are scarce [5, 6]. We use standardized questionnaires, protocols, equipment, and lab analysis methodology across sites making it possible to make these comparisons. However, we recognize a number of limitations when interpreting these results. The relative site sample size is small, and the age range of participants does not include the elderly who are at greatest risk of CVD. However, the narrow age range in the study allows for direct comparisons across site. Secondly, we present cross-sectional data and therefore cannot make inference on developing CVD. To overcome this, we use the Framingham risk score to estimate future CVD risk, which has been validated in several populations [28]. Our sampling strategy was not probabilistic and therefore it is not representative of the sites in terms of urban and rural locations. However, we aimed to select sites of broad differences in socioeconomic development and broad life style patterns, rather than probability samples representing the whole population. Finally, despite the training and use of standardized protocols, it is possible that some participants did not adhere to the study protocol and may have not fasted as instructed. This is a limitation to all population studies.

## Conclusions

Our data demonstrate the heterogeneity in the character and degree of emerging CVD risk among Afro-origin populations. This is one of the few studies that uses standardized data to measure CVD risk factors across sites in five countries spanning the socio-economic spectrum. Vital statistics data and measurement of CVD incidence rates are necessary before the disease burden can be described in any population. However, the correspondence between risk factors and future events is sufficiently well established to permit a clear prediction of what the emerging burden in these countries will be. Coronary heart disease rates are likely to remain low, as currently is the case in the Caribbean, [29] while stroke and the ravages of diabetes will be the greatest public health challenge. This is consistent with results from the Global Burden of Disease data where CVD deaths constituted only 8.8% of all deaths and 3.5% of all disability-adjusted life years (DALYs) in sub-Sahara Africa in 2010 [30]. Early detection and increasing access to pharmacologic treatment for hypertension could greatly reduce the burden from stroke. Repeated sample surveys, using standardized protocols and comparable data, at intervals of 10 years or less would provide extremely useful data from which the course of CVD disease can be predicted, thereby serving as the basis for policy decisions.

## Abbreviations

BMI: Body mass index
CVD: Cardiovascular disease
HDI: UN Human Development Index
HDL-C: HDL-cholesterol
LDL-C: LDL-cholesterol
METS: Modeling the Epidemiologic Transition Study
MPVA: Moderate vigorous physical activity by accelerometer
TC: Total cholesterol
TG: Triglycerides
